# “Know your medicines, know your health”—public perspectives on medicines and health awareness campaigns

**DOI:** 10.3389/fpubh.2025.1541185

**Published:** 2025-02-14

**Authors:** Faten Alhomoud, Kawthar Alameer, Zahra’a Almousa, Manar Almatar, Wafa Alzlaiq, Farah Kais Alhomoud, Dana Alsugeir

**Affiliations:** ^1^Department of Pharmacy Practice, College of Clinical Pharmacy, Imam Abdulrahman Bin Faisal University, Dammam, Saudi Arabia; ^2^Pharmacuetical Care Department, King Fahad Hospital of the University, Imam Abdulrahman Bin Faisal University, Dammam, Saudi Arabia; ^3^Pharmacy Services Department, Johns Hopkins Aramco Healthcare, Dhahran, Saudi Arabia

**Keywords:** medicine, health, health promotion, health education, public health, drug information services, campaign, patient education as topic

## Abstract

**Background:**

Irrational medication use is likely to result in therapeutic failure and disease progression. One way to increase public awareness about appropriate medication use is to design and deliver a public health medicine awareness campaign. This study aims to assess the public’s attitudes and experiences related to medication use and health awareness campaigns in Saudi Arabia.

**Methods:**

This is a cross-sectional survey study. Participants were aged 18 or over and able to speak Arabic or English. An online survey was distributed to a convenient sample of 451 participants by email and social media via an internet link, leading to a web-based survey platform in QuestionPro. Bivariate and multivariate analyses were used to assess the associations.

**Results:**

Three hundred and forty-seven participants (76% female, aged 18–85 years) were on a mean (SD) of two (1.86) prescription-only-medicines (POMs), and 225 were on non-prescription medicines. Seventy-one and 63 % of those surveyed consulted a doctor or a pharmacist, respectively, for advice about their medications. The participants were curious mainly about the side effects of treatment (79%), followed by drug interactions and contraindications (55%). Most participants agreed or strongly agreed that their medications were necessary to improve their condition (82%), prevent the progress of their condition (85%), and reduce the risk of complications (90%). Seventy-seven percent of participants reported seeing a public health campaign previously. TV (58%) and Twitter (55%) were reported as the most appropriate tools to help deliver a good public health campaign. Ninety-one percent believed that a public health campaign can increase people’s awareness about their lifestyle, and 73% declared that medication should be part of it.

**Conclusion:**

The findings can be a foundation for developing and implementing medicines and health awareness campaigns to enhance public understanding of health and medication use.

## 1 Introduction

Across the globe, the burden of chronic diseases is increasing. The World Health Organization (WHO) has estimated that 75% of the global population has at least one long-term condition (LTC) (1, 2). Almost one in four of the Saudi population has an LTC (3–6). Effective management of long-term conditions heavily depends on appropriate medication use; however, studies indicate that approximately 50% of patients with chronic diseases do not adhere to their prescribed medications (7–11). A study conducted in Saudi Arabia to examine the factors contributing to medicine-related problems (MRPs) among diabetes mellitus (DM) and cardiovascular diseases (CVDs) patients found that participants were not familiar with their medicines or how to use them effectively (7). Some patients could not name their medications or describe their shape. Similarly, they could not state the specific effect of each type of medicine, and their knowledge was restricted to their general indication (for instance, DM or CVDs) (7). Few patients were able to identify the side effects of their medicines. Some patients had a misunderstanding regarding the side effects of medicines, such as believing that the use of insulin can destroy the body. Others believed that exercise and diet could cure DM without using medicines to manage it (7). Consequently, treatment ineffectiveness was detected because patients did not take their medicines as prescribed. For example, patients reported blood sugar and blood pressure fluctuations, which they adjusted by changing medication doses or food intake without consulting a healthcare provider (7).

Failure to take medicines as intended is likely to result in disease progression and complications, therapeutic failure, and the need for more aggressive treatments, which might increase the risk of MRPs (8–12). Unnecessary suffering, loss in productivity, and even premature death can also result from poor adherence to medications (7–12). Avoidable medical expenses may also follow due to the waste of expensive medicines that are unused or misused and hospitalizations (8–12). One of the ways to address medication adherence challenges and promote rational medication use is the integration of medication-focused messages within public health campaigns. Public health campaigns can be defined as “organized communication activities designed to raise awareness, induce behavior change, and improve quality outcomes for individuals and populations” (13). These campaigns in Saudi Arabia have traditionally focused on preventing diseases and modifying behavioral risk factors, such as tobacco use, physical inactivity, and unhealthy diet (14, 15). While these initiatives have been widely implemented, limited attention has been given to medication use awareness as part of public health efforts. Given the essential role of medicines in managing chronic conditions, integrating medication education into public health campaigns could improve adherence and reduce co-morbidities, multiple drug therapies, medication-related problems (MRPs) (16). A systematic review by Ranjbar et al. (16) analyzed public health campaigns focusing on medication use awareness and found that such initiatives could positively impact medication adherence, reduce medication-related issues, and enhance public knowledge about safe medicine use. The review highlighted various educational strategies, including pharmacist-led interventions, video-based education, and community awareness programs, which were implemented across different countries (e.g., the US, UK, Taiwan, Canada, Jordan, Australia, and Malaysia), and healthcare settings (e.g., community pharmacies, hospitals, and public health initiatives). Some studies focused on general medication use, and others addressed specific use, such as antibiotic use and benzodiazepine use. These findings support the need for structured medication education within public health campaigns to optimize health outcomes and improve medication literacy.

Despite the recognized importance of medication literacy, there is a gap in research examining public perspectives on medication use within public health campaigns in Saudi Arabia. A previous study has explored the public’s knowledge of medication use in specific populations, such as patients with diabetes and cardiovascular disease (7), but a broader assessment of medication awareness in the general population remains unexplored. Furthermore, while campaigns promoting general health awareness are common (14, 15, 17), there is little evidence of structured medication-related campaigns in Saudi Arabia.

This study aims to fill this gap by assessing the public’s attitudes and experiences regarding medication use and public health awareness campaigns in Saudi Arabia. By understanding how the public engages with health campaigns and their perceptions of including medication-related messages, this research provides insights that could inform the development of targeted interventions to promote rational medication use and enhance public health outcomes.

### 1.1 Study objectives and hypotheses

The study aims to assess the public’s attitudes and experiences related to medication use and health awareness campaigns in Saudi Arabia. Specifically, the objectives of this study are:

1. To evaluate public awareness and knowledge regarding the use of prescription and non-prescription medications.
2. To examine the sources of information that individuals rely on when making decisions about medication use.
3. To assess the level of public engagement with health awareness campaigns and their perceived effectiveness.
4. To explore public perspectives on whether medication education should be integrated into public health campaigns.
5. To evaluate the relationship between individual characteristics (age, occupation, and prescription medication use) and public perceptions regarding the inclusion of medication use in public health campaigns.

The hypothesis of the study is:

There is a significant relationship between individual characteristics (age, occupation, and prescription medication use, etc.) and the belief that medication use should be part of public health campaigns.

## 2 Methods

### 2.1 Study design, setting, sampling, and data collection

A descriptive cross-sectional survey study was conducted in Saudi Arabia. Inclusion criteria were as follows: participants aged 18 or older and able to speak Arabic or English. Participants were excluded if they were non-residents of Saudi Arabia and did not complete the survey or provided incomplete responses. An internet-based survey questionnaire was distributed to a convenient sample of participants. The survey was hosted on the internet-based survey site QuestionPro, which enabled anonymous and secure participation (including consent to take part) and anonymous data collection. The QuestionPro site created a social media link and a web-based link to the survey, which were posted on the relevant social media forums (Twitter®, Facebook®, WhatsApp®) and web pages to evaluate the general public’s awareness of medication use and public health campaigns using Ranjbar’s questionnaire (18). Individuals were given an explanation with regard to the purpose of the study and were asked to provide informed written consent before filling out the online survey.

### 2.2 Study questionnaire

The Ranjbar questionnaire is a validated self-administered survey instrument developed at the Department of Clinical and Pharmaceutical Sciences, School of Life and Medical Sciences, University of Hertfordshire (18). It has previously been used in the United Kingdom. The questionnaire consists of three sections. Participants’ characteristics were collected in the first section, including age, gender, nationality, province of current residence, educational status, occupation, and health status. Section two consisted of ten close-ended questions to provide information about the awareness of medication use. This section explored participants’ understanding and behaviors regarding medication, including:

- Past experiences with prescribed and non-prescribed medications.
- Knowledge about their regular medications (e.g., name, purpose).
- Sources of advice and information regarding medications (e.g., healthcare providers, internet).
- Interest in specific information about prescribed medications (e.g., side effects, interactions, dependency).
- Participants are also asked to rate their agreement with statements related to the benefits of prescribed medications (e.g., improving conditions, prolonging life). A five-point Likert scale (1 = strongly disagree; 5 = strongly agree) was used to evaluate these beliefs.

The third section consisted of 10 open- and closed-ended questions and assessed awareness and engagement with public health campaigns, covering:

- Familiarity with and recollection of campaigns.
- Preferences for campaign delivery methods (e.g., TV, radio, social media, posters).
- Perceived effectiveness of campaigns in influencing lifestyle changes.
- Opinions on the inclusion of medicine use in public health campaigns.

Participants rated the effectiveness of public health campaigns on various topics, including healthy eating, physical activity, and weight management. These evaluations were made using a 5-point Likert scale, with responses ranging from “Excellent” (1) to “Very Poor” (5). Additionally, an optional “Not Applicable” (NA) response was provided for statements deemed irrelevant to the participants.

### 2.3 Validity and translation of the questionnaire

To ensure content validity, three expert pharmacists reviewed the questionnaire, which evaluated its clarity, applicability, thoroughness, and cultural sensitivity. Additionally, face validity was assessed by piloting the questionnaire with five individuals from the target population who were not part of the study population. They provided feedback on the comprehensibility and appropriateness of the questions. No major modifications were required. For linguistic validation, the questionnaire was translated from English to Arabic by a parallel blind translation technique (19) where two independent bilingual researchers translated the questionnaire separately; then, both versions were compared, and recommended amendments where necessary were discussed before being finalized. To further enhance accuracy, the questionnaire was then reviewed by a further Arabic-speaking pharmacist and pretested for content, readability, and comprehension in a group of five target language speakers. This method was chosen to minimize individual translator bias and ensure linguistic accuracy and conceptual equivalence.

### 2.4 Sample size

Because there have been no previous similar studies conducted in Saudi Arabia or other Gulf Cooperation Council (GCC) countries, the sample size was decided based on the assumption that the response proportions to most of the questions are 50%. The Raosoft sample size calculator was used to determine the sample size using a 95% confidence interval and a 5% margin of error. With a population size of 21,114,561 people aged ≥18 years old living in Saudi Arabia and an expected response of 50%, the minimum sample size required for this study was 385.

### 2.5 Data analysis

Data were exported from QuestionPro and were entered and analyzed using Statistical Package for the Social Sciences (SPSS 22, IBM Corp., Armonk, NY, United States). The survey was designed in a manner that required a response to all questions. Descriptive statistics were conducted for multiple-choice or close-ended questions, and continuous variables were presented as mean ± SD and categorical variables as frequencies and percentages based on the number of responses per question. For several items, the percentage values totaled above 100%, which indicated the participants gave multiple responses for one item (as allowed for certain items). Similar responses were grouped into like categories for open-ended questions, and then frequencies and percentages were calculated from the valid responses for each category. The results were also presented in tabular and graph forms. The records were double-checked and cleaned by three researchers.

Univariate analyses (frequency counts and percentages) were carried out to determine participants’ characteristics, public health campaigns, and medication use campaigns. Bivariate analysis was performed using the Chi-squared test to assess the association between participants’ characteristics and the public health and medication use campaigns. A logistic regression model assessed the association between participants’ characteristics (i.e., gender, age groups, education, occupation, province of current residence, prescription, and non-prescription medication use) and public health campaign and medication use campaign. Adjusted odds ratios plus 95% confidence intervals (CI) for the independent variables were calculated. Any participants’ characteristic with *p*-value ≤0.05 was taken as a significant predictor of the likelihood of believing that a public health campaign can increase people’s awareness about their lifestyle and whether “use of medicines” should be part of such a public health campaign.

Two logistic regressions were run, where the binary outcome variables were: (i) ‘Do you think a Public Health Campaign can increase people’s awareness about their lifestyle?’ (Yes or No); and (ii) ‘Do you think “use of medicines” should be part of a public health campaign?’ (Yes or No). Outcome variables for (i) and (ii) were re-coded so that data reporting ‘Do not know’ were assumed to be the equivalent of ‘No’. The explanatory variables for both regressions were gender, age, education, occupation, province of current residence, and prescription and non-prescription medication use (entered as categorical variables).

## 3 Results

### 3.1 Survey response and participants’ characteristics

Of the 868 participants who opened the survey, only 451 responded and completed it (response rate: 52%). The other 417 individuals viewed the survey but did not fill it out. The majority of participants were female (342/451; 76%), were Saudis (431/451; 96%) and were aged 18–24 years (157/451; 35%). The majority held above high school qualifications (305/451; 68%) and were either employed (full-time or part-time workers) (159/451; 35%) or students (144/451; 32%). The three regions where the most participants were located were Eastern (297/451; 66%), Central (69/451; 15%) and Western (61/451; 14%) provinces. Most participants had very good (209/451; 46%) and good health (201/451; 45%). A full overview of the participants’ characteristics is shown in Table 1.

**Table 1:** Characteristics of participants recruited into the study.

Parameter		Total sample (%) *n* = 451
Gender	Male	109 (24.2%)
	Female	342 (75.8%)
Age (years)	18–24	157 (34.8%)
	25–34	114 (25.3%)
	35–44	75 (16.6%)
	45–54	56 (12.4%)
	55–64	41 (9.1%)
	65–74	7 (1.6%)
	≥75	1 (0.2%)
Nationality	Saudi	431 (95.6%)
	Non-Saudi	20 (4.4%)
Education	Postgraduate	64 (14.2%)
	Undergraduate	225 (49.9%)
	High school	138 (30.6%)
	Intermediate school	5 (1.1%)
	Primary school	2 (0.44%)
	No qualification	1 (0.22%)
	Other	16 (3.54%)
Occupation	Employed as a full-time worker (≥ 30 h a week)	143 (31.7%)
	Employed as a part-time worker (< 30 h a week)	16 (3.5%)
	Self-employed	18 (4.0%)
	Unemployed	29 (6.4%)
	Retired	37 (8.2%)
	Homemaker	64 (14.2%)
	Student	144 (31.9%)
Province of current residence	Eastern province	297 (65.9%)
	Central province	69 (15.2%)
	Western province	61 (13.5%)
	Northern province	12 (2.7%)
	Southern province	12 (2.7%)
Health status	Very good	209 (46.3%)
	Good	201 (44.6%)
	Fairly good	38 (8.4%)
	Bad	1 (0.2%)
	Very bad	2 (0.4%)

### 3.2 The medication use awareness

Three hundred and forty-seven participants (76.9%) had taken prescription-only-medicines (POMs), and 225 (50.3%) had used non-prescription medications. The 347 participants reported using a mean of 2 POMs (SD 1.86, range 1–15). The majority of these participants (262/347, 75%) were taking ≤2 POMs.

When asked about whom they sought advice from if they had a query regarding their medications, 71 (322/451) and 63 (283/451) percent of those surveyed consulted a doctor or a pharmacist, respectively, for advice about their medications, followed by family members (104/451; 23%). Medication leaflets (267/451; 59%), pharmacies (261/451; 58%), and the Internet (240/451; 53%) were reported as major sources of medication information. Participants were asked to comment on what type of information would interest them when they took a medication prescribed to them by a healthcare professional (Table 2). Of the participants who responded, 355/451 (78.7%) were curious mainly about side effects of treatment, followed by drug interactions (247/451; 54.7%) and how the medication works in the body (232/451; 51.4%), and what will happen if a patient does not take the medication (207/451; 45.9%) or misses a dose of the medication (200/451; 44.4%).

**Table 2:** The type of information participants were interested in regarding their medicines.

Type of information	All sample (*n* = 451)
Its side effect	355 (78.7%)
Does it interact with food or other medication	247 (54.7%)
How it works in the body	232 (51.4%)
What will happen if I do not take it	207 (45.9%)
What will happen if I miss a dose	200 (44.4%)
Is there any better alternative	151 (33.5%)
Its effect in long-term use	147 (32.6%)
Does it cause dependency	144 (31.9%)
Nothing	24 (5.3%)
Other	7 (1.6%)

**Figure 1:**
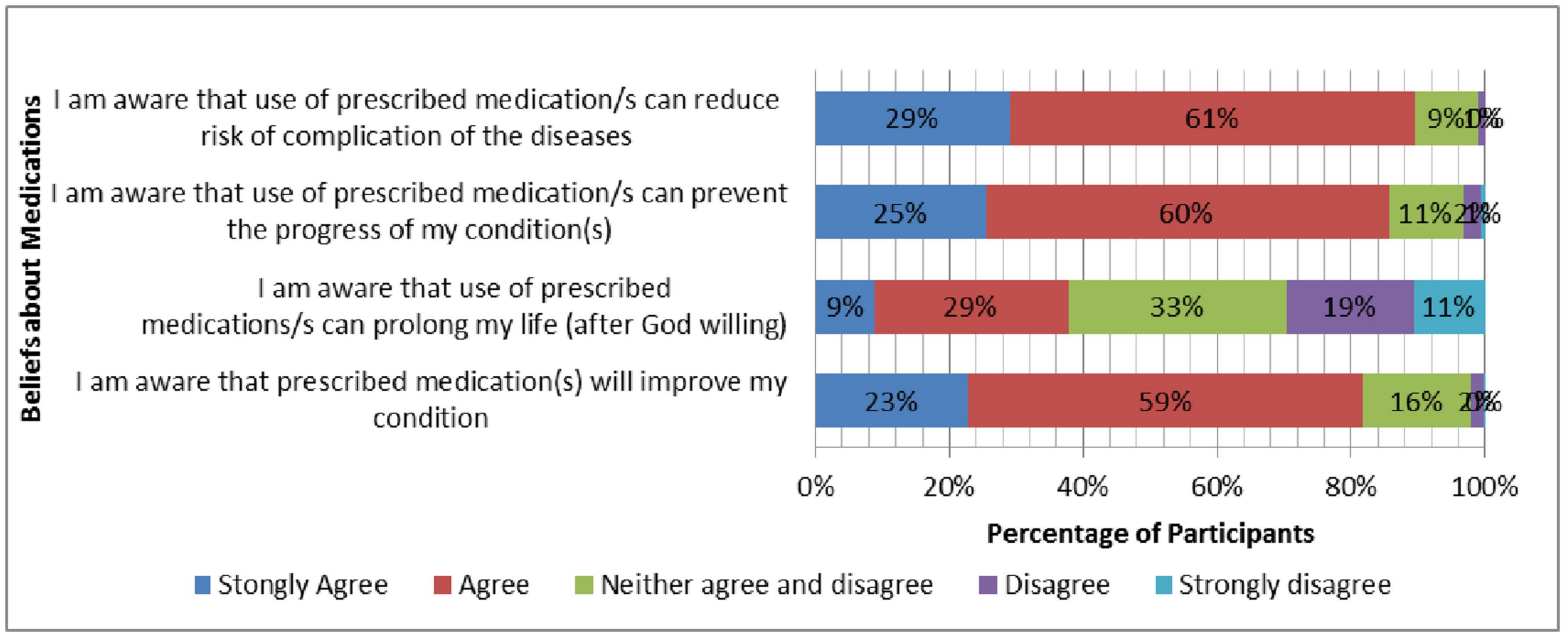
Participants’ responses (%) to questions related to beliefs about medicines.

Figure 1 illustrates the participants’ degree of agreement toward beliefs relating to the medication questions. The majority of participants responded that they either agreed (266/451; 59%) or strongly agreed (103/451; 23%) that their medication(s) were necessary to improve their condition. A larger proportion of participants either agreed (271/451; 60%) or strongly agreed (115/451; 25%) that their medication(s) can prevent the progress of their condition. More participants agreed (273/451; 60.5%) or strongly agreed (131/451; 29%) that the use of prescribed medication(s) can reduce the risk of disease complications. Nearly 40% of participants either agreed (130/451; 28.8%) or strongly agreed (40/451; 8.9%) that their prescribed medication(s) can prolong their life (after God willing). About 33% of participants neither agreed nor disagreed regarding whether their prescribed medication(s) can prolong their life.

### 3.3 The public health awareness

**Figure 2:**
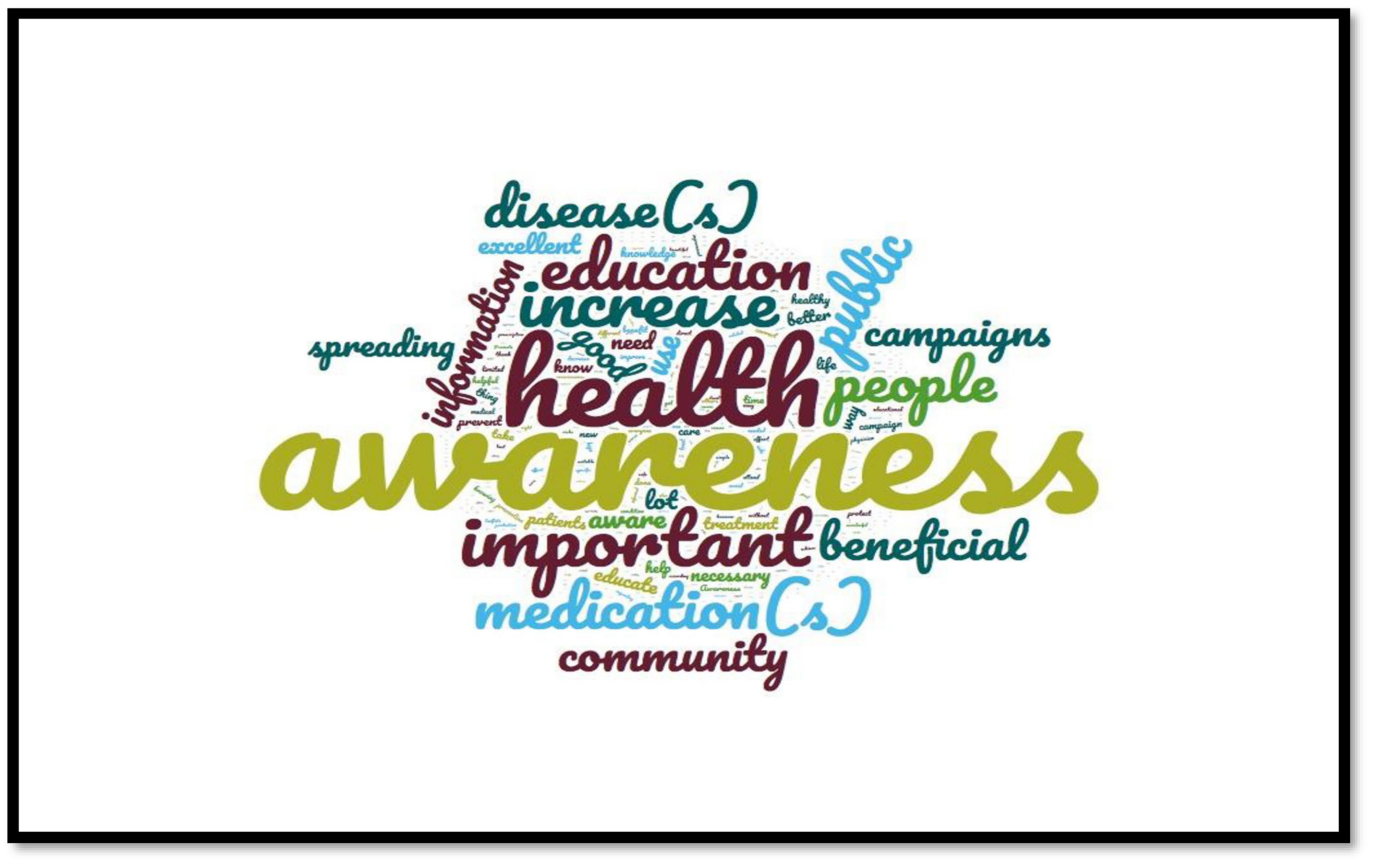
Most commonly repeated words revealed by participants on what public health campaigns meant to them.

When asked to summarize in three words what public health campaigns meant to them (Figure 2), participants used the following words: “awareness,” “health,” “important,” “increase,” “education,” “medication(s),” “people,” “public,” “disease(s),” “beneficial,” “community,” “information,” “campaign(s),” “good,” “spreading” and “use.”

Seventy-seven percent of participants (347/451) reported previously seeing a public health campaign. Nearly 50% (245/451; 54.3%) of participants were unaware of any public health campaign. Participants were asked to name some of the public health campaigns they remembered. The five most remembered public health campaigns reported by participants were breast cancer (86/207; 41.7%), followed by diabetes (63/207; 30.6%), healthy lifestyle (31/207; 15%), smoking (15/207; 7.3%) and hypertension (12/207; 5.8%).

Poster (284/451; 63%), leaflet (251/451; 55.7%), advertisement on TV (194/451; 43%), and Twitter (149/451; 33%) were selected as the most effective ways to make participants remember a public health campaign. TV (260/451; 57.7%), Twitter (246/451; 54.6%), poster (217/451; 48%), billboard (209/451; 46.3%), animation/pictures (156/451; 34.6%) and leaflet (150/451; 33.3%) were reported as the most appropriate tools to help in delivering a good public health campaign.

When asked whether a public health campaign helped participants change their lifestyle, 118 out of 451 participants (26%) responded in the affirmative. The four primary campaigns that helped participants to change their lifestyle were healthy lifestyle (52/118; 44%), diabetes (25/118; 21%), breast cancer awareness (22/118; 19%), and weight control (19/118; 16%) campaigns. Ninety-one percent (412/451) believed that a public health campaign can increase people’s awareness about their lifestyle, and 73% (330/451) declared that medication should be part of a public health campaign.

**Figure 3:**
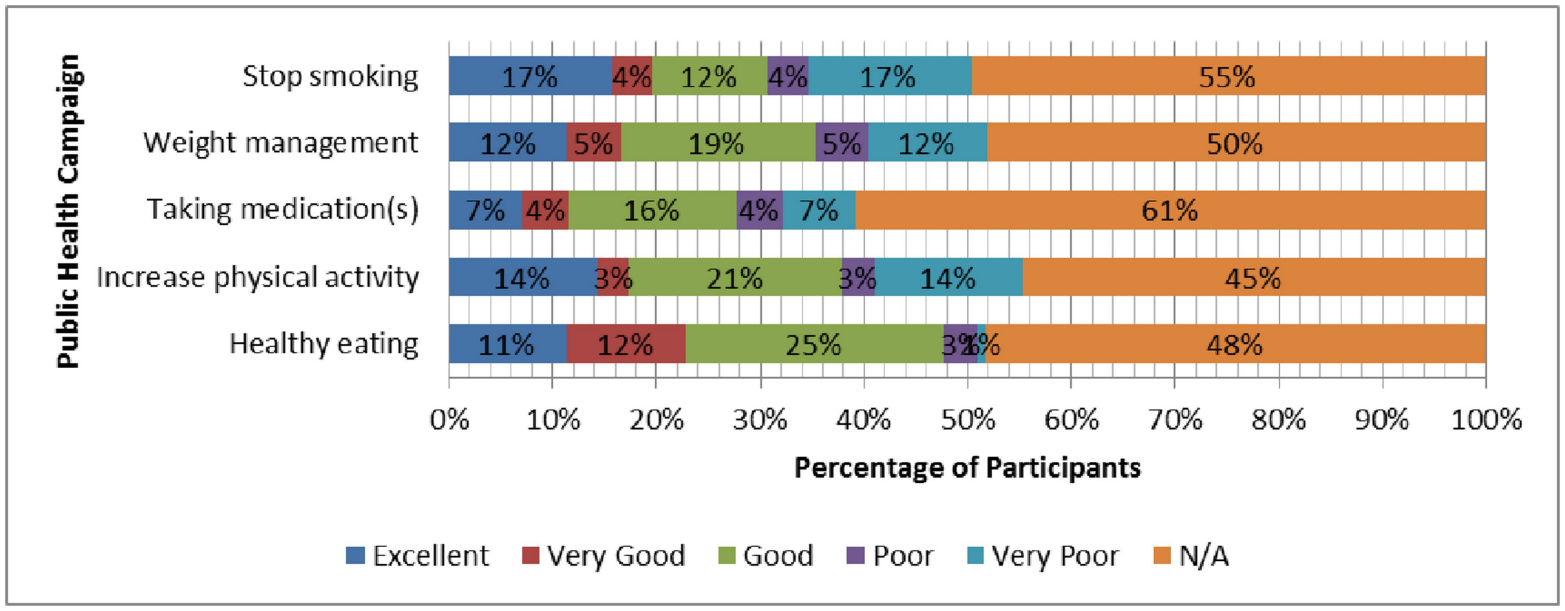
Participants’ responses (%) to questions related to rating levels of public health campaigns delivered on five different statements regarding campaign topics.

With regard to the statement on “healthy eating,” nearly 50% of participants (48%) rated the level of health campaign delivered as excellent (11%), very good (12%), or good (25%). Almost 40% of participants (38%) rated the level of health campaign delivered on “increase physical activity” as either excellent (14%), very good (3%) or good (21%). As for the taking medication statement, about one-quarter of participants (27%) rated the level of health campaign delivered on “medication taking” as either excellent (7%), very good (4%), or good (16%). Thirty-six percent of participants rated the level of health campaign delivered on “weight management” as excellent (12%), very good (5%) or good (19%). About 33% appreciated the level of health campaign delivered on “smoking cessation,” responding with either excellent (17%), very good (4%), or good (12%). Figure 3 shows the ratings for levels of public health campaigns delivered on five different statements regarding campaign topics.

### 3.4 Bivariate and multivariate analysis

There was no relationship between participants’ characteristics and their evaluation of public health awareness campaigns at bivariate analysis (see Table 3) or between participants’ characteristics and likelihood of believing that a public health campaign can increase people’s awareness about their lifestyle at multivariate analysis (see Table 4). The factors associated with participants’ perception of incorporating medication use as a part of public health campaigns at bivariate analysis were age group, occupation, and use of prescription medications (see Table 3). The participants’ characteristics associated with the likelihood of believing that “medication use” should be part of public health campaigns at multivariate analysis were age group, occupation, and use of prescription medications (see Table 4). Those who were younger were less likely to think that medication use should be part of a public health campaign, as were those who were unemployed or students and those who did not use a prescription medication.

**Table 3:** The factors of the participants’ characteristics and chi-square test results for medication use campaign and public health campaign, *n* = 451.

Variable		Frequency *n* (%)	Do you think “use of medicines” should be part of a public health campaign?					Do you think a public health campaign can increase people’s awareness about their lifestyle?				
			Yes (%)	No (%)	X^2^	Df	*P*-value	Yes (%)	No (%)	X^2^	df	*P*-value
Gender	Female	342 (75.8)	245 (71.6)	97 (28.4)	1.69	1	0.193	310 (90.6)	32 (9.4)	0.901	1	0.342
	Male	109 (24.2)	85 (78)	24 (22)				102 (93.6)	7 (6.4)			
Age groups	55 and above years	49 (10.9)	44 (89.8)	5 (10.2)	15.9	4	0.003	46 (93.9)	3 (6.1)	0.606	4	0.962
	18–24	157 (34.8)	100 (63.7)	57 (36.3)				142 (90.4)	15 (9.6)			
	25–34	114 (25.3)	84 (73.7)	30 (26.3)				104 (91.2)	10 (8.8)			
	35–44	75 (16.6)	57 (76)	18 (24)				69 (92)	6 (8)			
	45–54	56 (12.4)	45 (80.4)	11 (19.6)				51 (91.1)	5 (8.9)			
Education	Postgraduate	64 (14.2)	53 (82.8)	11 (17.2)	4.86	2	0.088	61 (95.3)	3 (4.7)	1.981	2	0.371
	Undergraduate	225 (49.9)	166 (73.8)	59 (26.2)				206 (91.6)	19 (8.4)			
	Other	162 (35.9)	111 (68.5)	51 (31.5)				145 (89.5)	17 (10.5)			
Occupation	Employed	177 (39.2)	142 (80.2)	35 (19.8)	16.1	3	0.002	162 (91.5)	15 (8.5)	1.039	3	0.792
	Unemployed	93 (20.6)	63 (67.7)	30 (32.3)				83 (89.2)	10 (10.8)			
	Retired	37 (8.2)	32 (86.5)	5 (13.5)				35 (94.6)	2 (5.4)			
	Student	144 (31.9)	93 (64.6)	51 (35.4)				132 (91.7)	12 (8.3)			
Province of current residence	Eastern Province	297 (65.9)	218 (73.4)	79 (26.6)	0.02	1	0.878	272 (91.6)	25 (8.4)	0.058	1	0.809
	Other provinces	154 (34.2)	112 (72.7)	42 (27.3)				140 (90.9)	14 (9.1)			
Prescription medication use	Yes	347 (64.1)	265 (76.3)	82 (23.7)	7.416	1	0.006	317 (91.3)	30 (8.7)	0.001	1	0.975
	No	104 (35.9)	65 (62.9)	39 (37.1)				95 (91.4%)	9 (8.6)			
Non-prescription medication use	Yes	227 (50.3)	170 (74.9)	57 (25.1)	0.688	1	0.407	213 (93.8)	14 (6.2)	3.558	1	0.059
	No	224 (49.7)	160 (71.4)	64 (28.6)				199 (88.8)	25 (11.2)			

**Table 4:** Multivariate analysis of the factors of the participants’ characteristics associated with medication use campaign and public health campaign.

Variable		Frequency *n* (%)	Do you think “use of medicines” should be part of a public health campaign?			Do you think a public health campaign can increase people’s awareness about their lifestyle?		
			AOR	95% CI	*P*-value	AOR	95% CI	*P*-value
Gender	Female	342 (75.8)	1.00^a^			1.00^a^		
	Male	109 (24.2)	1.085	0.605–1.944	0.785	0.471	0.278–1.806	0.709
Age groups	55 and above years	49 (10.9)	1.00^a^			1.00^a^		
	18–24	157 (34.8)	0.137	0.031–0.597	0.008	0.543	0.082–3.598	0.543
	25–34	114 (25.3)	0.232	0.064–0.846	0.027	0.815	0.159–4.179	0.806
	35–44	75 (16.6)	0.256	0.068–0.965	0.044	1.061	0.195–5.775	0.945
	45–54	56 (12.4)	0.379	0.107–1.340	0.123	0.902	0.176–4.610	0.901
Education	Postgraduate	64 (14.2)	1.00^a^			1.00^a^		
	Undergraduate	225 (49.9)	0.853	0.353–2.063	0.725	0.376	0.090–1.571	0.180
	Other	162 (35.9)	0.865	0.390–1.917	0.721	0.522	0.139–1.963	0.336
Occupation	Employed	177 (39.2)	1.00^a^			1.00^a^		
	Unemployed	93 (20.6)	0.518	0.292–0.916-	0.024	0.769	0.331–1.785	0.540
	Retired	37 (8.2)	1.577	0.573–4.342	0.378	1.620	0.354–7.408	0.534
	Student	144 (31.9)	0.449	0.272–0.744	0.002	1.019	0.461–2.251	0.964
Province of current residence	Eastern Province	297 (65.9)	1.00^a^			1.00^a^		
	Other provinces	154 (34.2)	0.885	0.549–1.427	0.617	0.821	0.400–1.688	0.593
Prescription medication use	Yes	347 (64.1)	1.00^a^			1.00^a^		
	No	104 (35.9)	0.526	0.330–0.839	0.007	1.013	0.465–2.207	0.975
Non-prescription medication use	Yes	227 (50.3)	1.00^a^			1.00^a^		
	No	224 (49.7)	0.838	0.552–1.272	0.407	0.523	0.264–1.035	0.063

AOR, Adjusted Odds Ratio.

a Reference category for the logistic regressions.

## 4 Discussion

The results of this study provide evidence about the public’s attitudes and experiences related to medication use and public health campaigns. Although many participants encountered campaigns passively (e.g., seeing them in passing), they did not actively engage with the campaigns.

Participants named different campaigns that they remembered and which had helped them change their lifestyles. These campaigns focused on raising awareness of the disease and modifying behavioral risk factors associated with increased risk of the disease, such as unhealthy diet and weight, physical inactivity, and tobacco use. None of these campaigns focused on medication use awareness.

The most commonly reported campaigns that were concerned about disease awareness included breast cancer and diabetes campaigns, possibly because breast cancer is the most common cancer affecting women in Saudi Arabia, and the number of women facing this disease continues to rise (20). Saudi Arabia also has one of the highest prevalences of diabetes in the world, with increased prevalence in recent years (21).

This study sheds light on the lack of available campaigns that raise people’s awareness of medication use in Saudi Arabia despite the high number of people who were on prescription and non-prescription medications involved in this study. Raising awareness about medications use has the potential to encourage the public to seek medication advice from pharmacists, a step towards empowering them in their medication-taking practices, helping them to take control of their health, improve their quality of life, decrease disease progression and their overall healthcare expenses, and reduce MRPs (13). A study conducted in Italy to examine the attitude and practice of parents towards non-prescription medicines use for their children found that more than half of parents used over-the-counter drugs without physician supervision since self-care might be favored by the opportunity to buy the drugs in the supermarket corner, where customers can choose medicines by themselves which might increase risk of MRPs (22). Previous studies have shown that antibiotic self-medication or purchase without a prescription is even more worrying (23–26). Indeed, antimicrobial resistance is an increasingly serious threat to global public health, and the misuse of antibiotics is accelerating the process. In addition, a high frequency of non-evidence-based prescriptions of antibiotics to treat upper respiratory tract infections at the primary care level has been reported (27, 28). This is particularly important because most upper respiratory tract infections are viral. Training pharmacists in communication skills to provide understandable arguments to explain and to educate the patient on the proper use of antibiotics might also help rationalize antibiotic use for upper respiratory tract infections.

Evidence also shows that if people have prior knowledge of and access to high quality use of medicine information, up to three-quarters of medicine-related hospitalizations could be prevented each year (29, 30). Quality use of medicine means selecting appropriate medicines if a medicine is required, choosing rational management options, and guaranteeing the patient and carers have the skills and knowledge to use medicines effectively and safely (29, 30). One of the ways to alter people’s behaviors and increase public awareness about the appropriate use of medication is by designing and delivering a public health medicine awareness campaign.

In the current study, 91% of participants believed that a public health campaign can increase people’s awareness about their lifestyle, and 73% believed that using medicines should be part of a public health campaign. The logistic regression analysis results highlight the important effect of several factors on the likelihood of thinking that the “use of medicines” should be part of a public health campaign. Those who were younger or those who did not use a prescription medication were less likely to think that medication use should be part of a public health campaign. A possible explanation for this result is that older people tend to take more medications than younger people as they are more likely to have more than one long-term condition (31–33). Since risks of adverse drug outcomes increase with an increasing number of medications (33), older adults may require more targeted counseling during medical visits or educational campaigns to raise awareness about their medication use.

Those unemployed or students were also less likely to think that medication use should be part of a public health campaign. This could be because the majority of those who were unemployed (71/93, 76%) were aged between 18 and 44 years. Younger people and students may have better Internet skills to locate health and medication information than older adults. Younger members of the population use computers in school or at work. At the same time, some older adults do not know how to work with technology or are interested in doing so. Thus, they ask for help to locate information on the Internet from their children, grandchildren, or close relatives. This could be one of the reasons why older adults lag behind the rest of the population in computer and internet use. This claim was also supported by the number of older adults aged ≥65 years (1.8%) who accessed the Internet and filled out the current study’s online survey.

Previous research shows that using knowledge transfer methodology through educational campaigns can lead to improvement in people’s knowledge, which may lead them to initiate behavioral changes (16, 34). Developing a public health campaign that supports appropriate medication-taking behaviors requires comprehensive planning by determining whether sufficient evidence suggests an inappropriate medication-taking practice exists within a certain population. After that, a development phase needs to be conducted that helps to identify objectives and the desired behavior changes or attitudes policy makers want to effect among the target population. Evaluating the success of a campaign is also required by determining whether the messaging results in the desired and intended behavior change (16).

A systematic review of the research on the impact of public health campaigns in raising patients’ awareness of medication use (i.e., adherence and/or knowledge of medications) pre- and post-campaign results showed that only four out of 12 studies of these public health campaigns showed a rise in the level of patients’ awareness of medication use in their post-campaign surveys (16). This is despite various tools being used to convey the messages, such as educational games, face-to-face approach, written information, and videos. The findings of the systematic review support the need for a comprehensive public health campaign that concentrates on the use of medicines. Public health campaigns could be effective when some essential issues are resolved, such as the clarity of the campaign’s message. Regardless of the tools used to deliver the campaign, if the message is not clear and difficult to understand, the individuals will not be able to adapt to the necessary behavior changes. Thus, the campaign messages need to be clear, easy to understand, and delivered correctly to the appropriate intended audience using numerous platforms to increase the messages’ outreach and help participants make what are essentially behavioral changes. Before expecting behavioral change from a community, it is important to devote adequate time to the campaign and information transfer to the population (16).

The current study highlighted an important role for healthcare providers, including doctors and pharmacists, in providing advice regarding medication inquiries for participants involved in this study. Participants frequently mentioned that the pharmacy was a major source of medication information. Although physicians could play a pivotal role in providing health promotion messages that are aligned with the patients’ needs when they are in contact with the patients (32), pharmacists may be the ideal healthcare workers to provide public education about the proper use of medicines. This is because pharmacists are specialized in pharmacotherapy, easily accessible healthcare providers, have frequent contact with the public, and are ideally positioned to convey health promotion messages (9, 35, 36).

These findings are consistent with evidence from previous research showing that pharmacists have improved patients’ medication adherence through their expertise and specialized training in medications (9, 35, 36). Additionally, pharmacists have been identified as trusted sources of medication information (9, 35, 36). Prior studies have also shown that educational interventions conducted by pharmacists seemed to increase the public’s knowledge about the safe and proper use of antibiotics and improve the public’s understanding of antibiotic resistance (37–39). The interaction may also help to foster patients’ trust towards pharmacists (37–43).

Discussion with participants in this study revealed that they were curious mainly about commonly encountered side effects and what to do if a side effect is experienced, followed by drug interactions with food and other medicines, how the medication works in the body, and what will happen if a patient does not take the medication or misses a dose of the medication. Thus, when designing a campaign, the campaign should focus on information tailored to each participant’s situation, needs and preferences. Some studies have shown information on the side effects of medication to be a priority for patients (44, 45). This suggests that healthcare providers could provide more information, assess patients’ understanding of potential side effects, and address any misunderstanding. It is well known that providing information to patients about prescribed medicines is vital for them to understand the benefits and risks of medication and to facilitate their appropriate use. However, providing basic information such as how and why to take medicine does not guarantee appropriate medication use; the information should be tailored to meet the individual’s requirements. Different levels of information may be required for people who are even prescribed the same medicines. Thus, the quality of the information is more important than the quantity. The quality of information refers to the extent to which individuals perceive that information has met their needs and that they are satisfied with the information provided (44).

TV, Twitter, posters, billboards, animation/pictures, and leaflets were reported in the current study as the most appropriate tools to help deliver a good public health campaign. Saudi Arabia ranked fourth in Twitter use after the United States of America (USA), Japan, and the United Kingdom (UK). This is consistent with other studies in the literature that have used different techniques and promotional tools to raise public awareness and promote engagement in medicine-related issues, such as videos, brochures, face-to-face education, educational games, and TV advertisements (16).

Antibiotic campaigns, for example, that have not used television tend to have no or less impact on public knowledge and attitude towards antibiotic use (46, 47). In contrast, other antibiotic campaigns in England, France, and Belgium (48–50) that have used television and have been repeated constantly for several years have led to improved public and professional attitudes towards antibiotic use. The French campaign was conducted annually from 2002 to 2007 and used television and radio advertising (48). A progressive reduction in antibiotic use was identified after each campaign using a time series analysis. For example, a 27% reduction in antibiotic use was reported in 2007 (48). Another campaign, ‘Moxy Malone’, was conducted in England for 2 years to reduce the amount of inappropriate antibiotic prescriptions by general practitioners (GPs). Local pharmacists and GPs were interviewed by local media, such as television and radio stations, to give some professional education and prescribing support. Local newspaper health columns were also targeted. The Moxy Malone campaign was associated with a 5.8% absolute reduction in antibiotic prescribing in the intervention group compared with the control group (50).

Reviews of research on social marketing campaigns, including online campaigns, suggest that they can influence people to change their behavior and influence policymakers (51, 52). The Internet especially represents an increasingly common source of health-related information (53–55). This result is consistent with our findings, which showed that 53% of participants reported using the Internet as a major source of medication information. Social media and the Internet allow the sharing and spreading of medicine-related information and allow discussion about different health and medicine-related topics. Thus, health organizations should consider social media within their communication strategy to promote the appropriate web use for medicine-related information seeking in the general population (53). The study findings showed that TV and Twitter are the most appropriate tools to help deliver a good public health campaign should be interpreted carefully because the current study participants were recruited online. There might be a digital divide or a gap between those who do and do not have access to digital information and communication technologies (ICT). For example, the Internet, like any technological innovation, primarily benefits younger and/or more educated people (53, 54). This was evident in the current study, as the vast majority of the sample were aged 18–34 and were educated.

Previous studies showed that younger people prefer to receive health information through the Internet or other electronic means (53, 54, 56), while older people prefer newspapers (56). In the future, preferences may be angled towards online options across various age groups (56). For now, a mixed approach is advised based on prior assessment of community preferences. One important consideration is that online campaigns can achieve higher reach at comparatively low cost (51).

Social media can increase engagement with healthcare issues, enable debate and discussion, and create virtual social networks (57, 58). However, there may be unintended consequences and risks. Concerns regarding misinformation, misinterpretation of health information, and potential difficulties with the confidentiality of personal information remain limitations of using the Internet and social media in delivering health- and medicine-related information, which may compromise health, medicine-taking behaviors, and health outcomes (53, 54, 59–61). In addition, there is a gap in the evaluation of digital platforms true effectiveness. A systematic review by Faus et al. (62) highlighted that while social networks such as Twitter offer high reach and low-cost dissemination, limited evidence supports their impact on actual behavioral changes in public health. Most evaluations focus on engagement metrics rather than assessing whether these campaigns improve health literacy, medication adherence, or long-term behavior modification. In the present study, we found that 55% of participants considered Twitter as an appropriate tool for delivering public health campaigns. Yet, a lack of structured evaluation methods to assess its influence on medication awareness and adherence remains. Future campaigns should integrate post-campaign behavioral assessments and longitudinal follow-ups to determine whether social media-based awareness efforts translate into meaningful health outcomes.

The findings of the current study and review of previous literature highlighted several lessons, some of which may be of general value when delivering a medication awareness campaign:

1. The campaign topic should not be selected according to the global health issues facing the world in a certain year; instead, it should be tailored to a specific country and its cultural environment for a message to be effective. By knowing what problems affect a specific country in a certain year regarding medication use or what behavior needs to be changed, a campaign can be designed to tackle these problems or influence behavioral changes. This can be done by reviewing previous literature to determine a country’s real and/or perceived needs.
2. Campaigns should contain brief, clear, and varied messages – ‘threatening’ and ‘supportive’ delivery styles to complement each other. For example, suppose the campaign is about high-risk medicines. In that case, a ‘threatening’ message can be about the implications of not taking these medicines as directed, whereas a ‘supportive’ message can be about fewer health-related absences from work, more quality time with family, fewer emergency room visits, fewer hospital admissions and getting the most benefit out of treatment if medications are taken as indicated.
3. Getting the messages out requires collaboration with television, radio stations, and local newspapers. In addition to using multiple medical channels, social media such as Twitter can spread medicine-related messages at a low cost and large reach. Other tools that can also help in delivering key messages are posters, billboards, animations/pictures, educational games, and leaflets. This will increase the outreach of the key messages to the whole community.
4. Educating the public should include both verbal and written information. The written materials should use plain language at the fifth-grade level or lower. Materials for health education need to be simple and easy to understand for people of all literacy levels. Providing simple, accessible materials could empower patients to take great control over their health, lower their fear of disease and treatment, and allow them to make more active decisions about their health (45).
5. Besides the content and type of information, the timing and moment of providing key messages are also vital. For example, conducting a campaign during International Patient Safety Week, which starts on September 17th, is an option. The campaign should be repeated at the same time each year, and its key messages should be changed or updated every year based on community needs and wants. As described earlier, breast cancer and diabetes are growing public health concerns in Saudi Arabia. So, if the campaign aims to raise people’s awareness about diabetic medication, the campaign could be delivered on November 14th, which is World Diabetes Day, but if the campaign aims at educating the audience about breast cancer medication, October is the best month to conduct such a campaign as it is Breast Cancer Awareness Month.
6. Preferably, the focus of the campaign should be on increasing awareness about either a single group of drugs, such as appropriate use of benzodiazepines, or a group of drugs that treat the same condition, such as antibiotics, antiretroviral therapy, anticoagulants, diabetic and antihypertensive medications, etc. Suppose a patient has a case-specific question that is not within the scope of the campaign. In that case, the pharmacist can direct that patient to a specialized pharmacist in the healthcare sector or another healthcare provider who can answer his/her specific question. The campaign topic can also be a behavior that HCPs and policymakers want to change for the better, such as increasing patient engagement or improving patient adherence etc. Campaigns can also be about medicine optimization, encouraging people to visit pharmacies to learn more about their medicines and take advantage of pharmacy services and expertise.

### 4.1 Strengths, limitations, and future research

Strengths: (1) this study highlighted the area where very little research has been conducted as regards measuring awareness of public health and medication use campaigns in Saudi Arabia; (2) using an online survey technique in the current study was ideal to obtain a good sample size and sample people who live in different regions of Saud Arabia. Limitations: (1) the sample of this research included mainly Saudi nationals; therefore, results may not be transferrable to non-Saudis; (2) the participants of the current study had to be computer literate to answer the survey questions online, which may lead to sampling bias; (3) there was also a difficulty reaching certain types of participants, such as those who do not have internet access (e.g., the older adult and people who reside in remote areas); (4) although one of the questions in the survey aimed to assess participants’ beliefs about medicines in general, the responses provided by those who were not on any prescribed or non-prescribed medicines may not be valid. However, this limitation does not influence our main study finding (i.e., attitudes related to public health campaign or medication use campaign); (5) the fifth restriction is linked to the adopted cross-sectional survey design, whereby claims about the directionality of causal relationships between the dependent and independent variables cannot be verified; (6) the last limitation is that a reliability assessment of the questionnaire was not conducted. This decision was made because the questionnaire primarily included binary (Yes/No) and categorical multiple-choice items, which do not align with conventional reliability measures such as Cronbach’s Alpha. Since the study focused on obtaining descriptive responses rather than assessing an underlying construct, internal consistency analysis was not deemed appropriate. However, future studies may explore reliability testing for specific subscales where applicable. Future studies should also focus on designing a campaign to raise people’s awareness of the importance of using medicines safely and effectively to initiate behavioral changes. If successful, implementing such a campaign can shift the entire country’s behavior toward the appropriate use of medicines (16). Before being implemented nationally, the campaign should be evaluated for its reach, efficacy, effectiveness, and sustainability of impact in terms of awareness and behavior change.

## 5 Conclusion

In conclusion, the results of this study provide evidence about the public’s attitudes and experience related to medication use and public health campaigns. Medication use awareness campaigns might be needed to control irrational habits such as self-medication, lending, borrowing or using leftover prescription and non-prescription medications, and other irrational medication-taking behaviors. The findings of this study can be used as a basis for designing and delivering public health campaigns that raise medication awareness among the population in order to encourage responsible behavior in relation to prescribed and non-prescribed medications.

## Data Availability

The original contributions presented in the study are included in the article/supplementary material, further inquiries can be directed to the corresponding author.
